# Alcohol Consumption Patterns among Adolescents are Related to Family Structure and Exposure to Drunkenness within the Family: Results from the SEYLE Project

**DOI:** 10.3390/ijerph111212700

**Published:** 2014-12-08

**Authors:** Erik Rüütel, Merike Sisask, Airi Värnik, Peeter Värnik, Vladimir Carli, Camilla Wasserman, Christina W. Hoven, Marco Sarchiapone, Alan Apter, Judit Balazs, Julio Bobes, Romuald Brunner, Paul Corcoran, Doina Cosman, Christian Haring, Miriam Iosue, Michael Kaess, Jean-Pierre Kahn, Vita Poštuvan, Pilar A. Sáiz, Danuta Wasserman

**Affiliations:** 1 Estonian-Swedish Mental Health and Suicidology Institute, Tallinn University Social Work Institute, Tallinn 11615, Estonia; E-Mails: sisask.merike@gmail.com (M.S.); varnik.airi@gmail.com (A.V.); peeterv@suicidology.ee (P.V.); 2 Justice College, Estonian Academy of Security Sciences, Tallinn 12012, Estonia; 3 National Centre for Suicide Research and Prevention of Mental Ill-Health (NASP) at Karolinska Institutet, Stockholm SE-171 77, Sweden; E-Mails: v.carli@mclink.it (V.C.); Danuta.Wasserman@ki.se (D.W.); 4 Department of Child and Adolescent Psychiatry, Columbia University-New York State Psychiatric Institute, New York, NY 10032, USA; E-Mails: WassermC@childpsych.columbia.edu (C.W.); hoven@childpsych.columbia.edu (C.W.H.); 5 Medicine and Health Science Department, University of Molise, Via De Sanctis, 86100 Campobasso, Italy; E-Mails: marco.sarchiapone@me.com (M.S.); miriam.iosue@gmail.com (M.I.); 6 Feinberg Child Study Center, Schneider Children’s Medical Center, Tel Aviv University, Tel Aviv 69978, Israel; E-Mail: eapter@clalit.org.il; 7 Vadaskert Child and Adolescent Psychiatric Hospital, Budapest 1021, Hungary; E-Mail: judit.agnes.balazs@gmail.com; 8 Institute of Psychology, Eötvös Loránd University, Budapest 1064, Hungary; 9 Department of Psychiatry, School of Medicine, University of Oviedo, Centro de Investigación Biomédica en Red de Salud Mental (CIBERSAM), Oviedo 33003, Spain; E-Mails: bobes@uniovi.es (J.B.); frank@uniovi.es (P.A.S.); 10 Section for Disorders of Personality Development, Department of Child and Adolescent Psychiatry, Centre for Psychosocial Medicine, University of Heidelberg, Heidelberg 69047, Germany; E-Mails: Romuald.Brunner@med.uni-heidelberg.de (R.B.); michael.kaess@med.uni-heidelberg.de (M.K.); 11 National Suicide Research Foundation, Cork, Ireland; E-Mail: pcorcoram@ucc.ie; 12 Clinical Psychology Department, Iuliu Hatieganu University of Medicine and Pharmacy, Cluj-Napoca 400012, Romania; E-Mail: doina.octaviancosman@hotmail.com; 13 Research Division for Mental Health, University for Health Science, Medical Informatics Technology (UMIT), Hall in Tyrol 6060, Austria; E-Mail: Christian.haring@tilak.at; 14 Department of Psychiatry and Clinical Psychology, Centre Hospitalo-Universitaire CHU de NANCY, Université de Lorraine, Nancy 54500, France; E-Mail: jp.kahn@chu-nancy.fr; 15 Slovene Center for Suicide Research, UP IAM, University of Primorska, Koper SI-6000, Slovenia; E-Mail: vita.postuvan@upr.si

**Keywords:** alcohol, adolescent, risk-behaviour, family structure, family member drunkenness, SEYLE

## Abstract

There is expedient evidence showing that differences in adolescent alcohol consumption and other risk-behaviour depend on both family structure and family member drunkenness exposure. Data were obtained among adolescents (N = 12,115, mean age 14.9 ± 0.89) in Austria, Estonia, France, Germany, Hungary, Ireland, Israel, Italy, Romania, Slovenia and Spain within the European Union’s 7th Framework Programme funded project, ‘Saving and Empowering Young Lives in Europe (SEYLE)’. The current study reveals how adolescents’ alcohol consumption patterns are related to their family structure and having seen their family member drunk. The results revealed statistically significant differences in adolescent alcohol consumption depending on whether the adolescent lives in a family with both birth parents, in a single-parent family or in a family with one birth parent and one step-parent. The study also revealed that the abstaining from alcohol percentage among adolescents was greater in families with both birth parents compared to other family types. The study also showed that the more often adolescents see their family member drunk the more they drink themselves. There is no difference in adolescent drinking patterns whether they see their family member drunk once a month or once a week. This study gives an insight on which subgroups of adolescents are at heightened risk of alcohol abuse and that decrease of family member drunkenness may have positive effects on the drinking habits of their children.

## 1. Introduction

The consumption of alcohol is among the core risk behaviours among adolescents [1,2,3]. Alcohol can be a part of the adolescents maturing process and also a steppingstone towards harder substance abuse [4,5,6,7]. Alcohol consumption makes adolescents vulnerable to the occurrence of maladaptive behaviour, delinquency, violence, accidents, emotional instability, depression, social exclusion and suicide [8,9,10,11]. Alcohol consumption is not only deleterious to adolescent mental health and safety but also constitute a substantial economic burden to governments [12,13,14]. Despite obvious risks and adverse outcomes, alcohol consumption is still increasing among adolescents in some European countries [15,16,17].

### 1.1. Adolescent Alcohol Consumption

Children recognize alcoholic beverages and develop an attitude towards alcohol from as early as pre-school [18]. In 1995 a major international investigation, the European School Survey Project on Alcohol and other Drugs (ESPAD), on potential risk behaviours among adolescents revealed that adolescents in Northern European countries reported the highest levels of heavy drinking and intoxication [19]. Another major international study on the Health Behaviour of Schoolchildren (HBSC) revealed that weekly alcohol use and (early) drunkenness was increasing substantially with age (especially between ages 13 and 15) for boys and girls in all European countries. HBSC findings showed that the gender gap of different alcohol consumption has also declined between 1998 and 2006 [20].

Different types of adolescent alcohol consumption categories like heavy episodic drinking [17,21] and risky drinking [22,23] have been used by researches for more precise analysis of the risk-behaviour and its adverse consequences. The number of episodes of intoxication prior to age 16 has been found to be a strong predictor of adult alcohol problems [24].

### 1.2. Family Structure

Adolescence, as being a transitional stage from childhood to young adulthood, is accompanied by changes in the biological, psychological and social aspects of life [25]. The change constitutes in the imitation of adult behaviours [26,27], emotions and thought processes, new ways of dealing with wins and losses and experimenting with new coping mechanisms [28,29], which is only a small part of the internal and external changes of adolescents, which involve their entire identity. Many risk behaviours get their start from such innocent experimentations and imitations. The research on family structure’ effects on adolescents’ deviant behaviours such as delinquency, alcohol, cigarette and drug use have led to opposing results. Some studies have found no relationships between family structure and any adolescent deviant behaviour expressions what so ever [30], but others have constituted that adolescents living with both birth parents engaged less frequently in heavy alcohol use [31] and deviant behaviour [32] than those living in any other arrangements.

There is predominant evidence pointing towards the differences in adolescent alcohol consumption rates and other risk-behaviour depending on the family structure [33,34,35]. Research has linked the adolescent alcohol consumption to social and individual predicators as well as family and peer relationships [29,36]. While there is a shift in emotional attachment during early adolescence and an increase in the importance of peer approval [37,38], there is still evidence to support the continuing influence of parents on adolescent development in general [39] and the development of values [40] through late adolescence and into early adulthood [38,41].

Various studies have shown that, compared with children brought up in intact families with two birth parents, children whose family structure is different (single parent and one step-parent families) are more likely to have emotional and psychological difficulties and behavioural problems [33,42,43,44,45]. The prevalence of aberrant behavioural and emotional symptoms is lowest in children living with both their birth parents and highest amongst children living away from their birth parents [46], revealing no significant differences between single parent families and families with one step-parent [33]. Children in single parent families and families with one step-parent are at a disadvantage, in cognitive, emotional and behavioural terms, compared with those in two birth-parent families [33,47].

### 1.3. Family Structure, Family Member Drunkenness Exposure and Adolescent Alcohol Consumption

Adolescents’ immediate family and more specifically parents are usually the facilitators and imposers of social norms, overseeing the descending behaviour of adolescents towards alcohol. The manner in which parents regard adolescent alcohol consumption influences adolescents’ alcohol initiation and possible transition to heavier drinking [48]. Adolescent alcohol consumption is not only a result of family dysfunctions and unresolved physical or emotional development, but also a learned coping mechanism [49,50,51]. Conformably with any socially learned behaviour the rise of adolescent’ alcohol consumption is connected with witnessing family members’ corresponding behaviour. Hutchinson *et al*. [52] and Hayes *et al*. [48] have found that parents influence adolescents via their attitudes to drinking and, more directly, through the modelling of alcohol use. Bonomo *et al*. [53] reported that adolescents, who were exposed to alcohol consumption by a family member, are prone to initiate alcohol use earlier and engage in problem drinking at a younger age than non-exposed children. Alati *et al*. [54] showed that maternal drinking (more than one glass of alcohol a day), assessed when the adolescent offspring were at age 14, was a strong predictor of the adolescents’ concurrent alcohol problems at the age of 21. Although genetic and environmental factors play an important role in the formation of a young adult, social learning seems to determine a substantial amount of the outcome.

The search for contributing factors to adolescent alcohol consumption is important for developing social strategies and action plans to target necessary problem criteria. The aim of this study is to show adolescents’ alcohol consumption patterns depending on the family structure, and also to reveal the impact of a family member drunkenness exposure on adolescents’ alcohol consumption.

## 2. Methods

The 7th Framework European Commission funded project, Saving and Empowering Young Lives in Europe (SEYLE) is a Randomized Controlled Trial (RCT) evaluating preventive interventions for risk-behaviours among adolescents in Austria, Estonia, France, Germany, Hungary, Ireland, Israel, Italy, Romania, Slovenia and Spain with Sweden as the coordinating site. The data for this study was collected during the baseline assessment of the SEYLE project.

### 2.1. Subjects and Instrument

All SEYLE questionnaires were administered in the official language(s) of the specific country. In each country, a list of all eligible schools, within the study sites, was generated according to specific inclusion and exclusion criteria [55]. Schools were randomly selected to participate in SEYLE. To meaningfully interpret the potential representativeness of each site, key parameters such as mean age, number of immigrants, population density, net income and gender proportion for each site were compared to the corresponding national data. Data at the national and local levels were extracted from Eurostat [56]. Ethical approval was obtained from the local ethical committees at each study site. Out of the 14,115 students who consented to participate, 1,720 were absent the day of the survey. This resulted in a total of 12,395 students who completed the questionnaire. An additional 83 subjects were excluded based on missing relevant data and after listwise deletion of the families with only grandparents or foster home or something else (n = 197; 1.6%) the total sample of 12,115 adolescents was included in the analyses (F/M: 6714(55.4%)/5401(44.6%); mean age: 14.9 ± 0.89). Sample variation by country was minimal (mean 1101.36; range 956:1426) so no adjustments were made. The SEYLE base-questionnaire gathered information on the (I) family structure and (II) alcohol consumption patterns of adolescents and also the (III) family member drunkenness exposure to adolescents.

### 2.2. Operationalization of Concepts and Statistical Procedures

From the perspective of **(I) family structure** the study assessed the answer to the question ‘*where you live permanently or most of the time and write down the people who live with you at your home*’ in 8 categories: mother, father, stepmother with father, stepfather with mother, grandmother, grandfather, foster home or something else with the availability to make multiple choices. For analysis the answers were combined into three family type categories disregarding any other family settings: (1) **both parents family**—birth father and birth mother in the family; (2) **single parent family**—one birth parent alone, either father or mother; and (3) **step parent family**—one birth parent (either father or mother) and one step-parent (either stepfather or stepmother) [33,57]. Families with grandparents living together with the parent(s) (n = 1580 [13%]) were included within the immediate family structure and not differentiated in this research.

The adolescent **(II) alcohol consumption patterns** in SEYLE base questionnaire were measured with 3 distinct questions (a) **drinking frequency**—‘*How often do you have a drink containing alcohol? For example, 0.33 l beer or cider; glass of wine or 4 cl of strong alcohol*’, (b) **drinking quantity**—‘*How many drinks containing alcohol do you have on a typical day when you are drinking?*’, (c) **drunkenness frequency**—‘*During your life, how many times did you drink so much alcohol that you were really drunk?*’*.* The answers to the question (a) were regrouped for analysis from 7-scale into 4-scale: never; once a month or less; 2 to 4 times in a month; 2 or more times in a week. The question (b) was regrouped from 5-scale into 4-scale: I never drink alcohol; 1 or 2; 3 or 4; 5 or more drinks. The question (c) remained in their original 4-scale: never; 1 or 2 times; 3 to 9 times; 10 or more times, and was analysed as such. For logistic regression analysis the answers to all three questions were regrouped also dichotomously: never *versus* 1 or more according to the question.

The variable **(III) family member drunkenness exposure** was measured with one question ‘*Have you ever seen a family member when they are drunk?*’. The possible answers were grouped as follows: no; sometimes; occasionally (*i.e.*, once a month); frequently (*i.e.*, once a week, every day).

Data analyses were performed with SPSS 17.0. The relationship between family structure and alcohol consumption was measured in this research by χ²-test and the model of family member drunkenness exposure and adolescent drinking patterns depending on family structure was investigated by logistic regression analysis. The level of statistical significance was set at α = 0.05.

## 3. Results

### 3.1. Frequencies of Family Structure and Adolescent Drinking Patterns

The frequency distribution of family structure groups based on the participating 11 countries revealed that 78.2% (n = 9478) of the adolescents were from both parent families, 14.8% (n = 1789) from single parent families and 7.0% (n = 848) from step parent families. 36.0% of the adolescents reported never drinking alcohol, 33.1% reported drinking once a month or less, 22.7% 2 to 4 times a month, 8.2% 2 or more times a week. On the subject of drinking quantity, 37.5% reported—never drinking alcohol, 39.6% having 1 to 2 drinks, 13.5% 3 to 4 drinks, 9.4% 5 or more drinks per occasion. Regarding drunkenness frequency, 63.6% reported never having been really drunk, 22.1% reported having been really drunk 1 to 2 times in life, 9.8% 3 to 9 times in and 4.5% 10 or more times in life.

**Table 1:** Frequencies of categories describing adolescent drinking patterns in different family structure types.

			Family Structure							
			Both Parents Family		Single Parent Family		Step Parent Family		Chi-Square	*p*-Value
			**n**	**%**	**n**	**%**	**n**	**%**		
Adolescent drinking patterns										
	Drinking frequency									
		Never	3595	**38.3%**	515	29.1%	217	25.7%	114.78	<0.001
		Once a month or less	3069	32.7%	607	**34.3%**	294	**34.9%**		
		2 to 4 times a month	2018	21.5%	467	26.4%	235	27.9%		
		2 or more times a week	711	7.5%	181	10.2%	97	11.5%		
	Drinking quantity									
		I never drink alcohol	3724	**39.8%**	544	30.9%	215	25.6%	129.43	<0.001
		1 or 2	3651	39.0%	703	**40.0%**	378	**45.1%**		
		3 or 4	1161	12.4%	311	17.7%	146	17.4%		
		5 or more	821	8.8%	200	11.4%	100	11.9%		
	Drunkenness frequency									
		Never	6246	**66.5%**	970	**54.9%**	420	**49.8%**	194.01	<0.001
		1 or 2	1967	20.9%	464	26.2%	225	26.7%		
		3 to 9	836	8.9%	206	11.7%	130	15.4%		
		10 or more times	347	3.7%	128	7.2%	68	8.1%		

Note: **Bold**—prevalent subgroup.

### 3.2. Family Structure and Adolescent Drinking Patterns

Analysis revealed a statistically significant (*p* < 0.001) difference between defined family structure groups and adolescent drinking frequency (Table 1). More detailed investigation in pairs showed statistically significant (*p* < 0.001) differences in adolescent alcohol consumption frequency between both parent and single parent families and also between both parent and step parent families. Between single parent and step parent families the study revealed no statistically significant (*p* = 0.295) differences (Table 1). Logistic regression model showed that in single parent families (OR = 1.481) and step parent families (OR = 1.745) the odds of higher adolescent drinking frequency were statistically significantly (*p* < 0.001) greater, compared to both parent families (Table 2).

The comparison of adolescent drinking quantity showed statistically significant (*p* < 0.001) differences between all three family structure types (Table 1). Distinguishing the family structure types in pairs, the differences in adolescent drinking quantity remained statistically significant between both parent and single parent families (*p* < 0.001), both parent and step parent families (*p* < 0.001) and also between single parent and step parent families (*p* = 0.027). Logistic regression analysis revealed that that adolescents’ odds to have higher drinking quantities increased in single parent families (OR = 1.428) and in step parent families (OR = 1.823) statistically significantly (*p* < 0.001) compared to both parent families (Table 2).

Analysis of drunkenness frequency showed statistically significant (*p* < 0.001) differences between all three family structure types (Table 1). We found statistically significant differences by comparing the percentages of different adolescent drunkenness frequencies within family structure types in pairs—both parent families were statistically significantly different compared to single parent families (*p* < 0.001) and also to step parent families (*p* < 0.001). Adolescent drunkenness frequency in single parent families revealed to be statistically significantly (*p* = 0.022) different from step parent families (Table 1). Logistic regression analysis showed statistically significant (*p* < 0.001) increase of odds of adolescent drunkenness frequency in single parent families (OR = 1.601) and in step parent families (OR = 2.016) compared to both parent families (Table 2).

### 3.3. Family Member Drunkenness Exposure and Family Structure

Out of the sample of 12,115 46.8% (n = 5614) of adolescents reported never seen their family member drunk, 44.6% (n = 5348) have seen their family member drunk sometimes, 4.0% (n = 476) once a month, 2.7% (n = 321) once a week and 1.9% (n = 230) every day. In both parent families 48.3% adolescents reported never seeing their family member drunk compared to 43.9% in single parent families and 37.1% in step parent families.

**Table 2:** Logistic regression models on adolescent drinking patterns’ associations with family structure types and familial drunkenness exposure.

		Drinking Frequency				Drinking Quantity				Drunkenness Frequency			
		Never *vs*. once a month or more		95% Cl for Exp (B)		Never *vs.* 1 or more drinks		95% Cl for Exp (B)		Never *vs.* 1 or more times drunk		95% Cl for Exp (B)	
Variable	Level	OR	*p*-value	Lower	Upper	OR	*p*-value	Lower	Upper	OR	*p*-value	Lower	Upper
Constant			<0.001				<0.001				<0.001		
Age		1.647	<0.001	1.574	1.725	1.662	<0.001	1.588	1.740	1.724	<0.001	1.648	1.804
Gender		0.874	0.001	0.809	0.945	0.901	0.008	0.834	0.974	0.796	<0.001	0.737	0.861
Country		0.936	<0.001	0.924	0.947	0.913	<0.001	0.902	0.925	0.942	<0.001	0.930	0.954
**Family** structure (Base = Both parents family)	Single parent family	1.481	<0.001	1.322	1.660	1.428	<0.001	1.275	1.598	1.601	<0.001	1.438	1.781
	Step parent family	1.745	<0.001	1.481	2.056	1.823	<0.001	1.546	2.150	2.016	<0.001	1.740	2.335
Age		1.662	<0.001	1.587	1.742	1.679	<0.001	1.603	1.759	1.767	<0.001	1.686	1.851
Gender		0.868	<0.001	0.802	0.939	0.893	0.005	0.826	0.966	0.784	<0.001	0.724	0.850
Country		0.936	<0.001	0.924	0.948	0.912	<0.001	0.901	0.924	0.938	<0.001	0.926	0.951
**Familial drunkenness exposure** (Base = Have never seen a family member drunk)	Sometimes	1.957	<0.001	1.804	2.123	1.997	<0.001	1.841	2.166	2.661	<0.001	2.446	2.896
	Once a month	3.457	<0.001	2.722	4.390	3.457	<0.001	2.731	4.377	5.014	<0.001	4.104	6.125
	Once a week or more often	2.611	<0.001	2.123	3.210	2.329	<0.001	1.907	2.843	3.633	<0.001	3.023	4.367

**Table 3:** Frequencies of familial drunkenness exposure and adolescent drinking patterns.

			Seen Family Member Drunk (Familial Drunkenness Exposure)									
			Never		Sometimes		Once a month		Once a week or more		Chi-Square	*p*-value
			n	%	n	%	n	%	n	%		
Adolescent drinking patterns (habits)												
	Drinking frequency											
		Never	**2502**	**44.7%**	1569	29.5%	91	19.4%	131	23.9%	582.06	<0.001
		Once a month or less	1805	32.3%	**1840**	**34.6%**	**159**	**33.8%**	148	27.0%		
		2 to 4 times a month	1000	17.9%	1406	26.4%	142	30.2%	**156**	**28.5%**		
		2 or more times a week	283	5.1%	508	9.5%	78	16.6%	113	20.6%		
	Drinking quantity											
		I never drink alcohol	**2594**	**46.5%**	1619	30.5%	95	20.2%	149	27.4%	532.07	<0.001
		1 or 2	2109	37.8%	**2197**	**41.3%**	**201**	**42.6%**	**214**	**39.3%**		
		3 or 4	547	9.8%	885	16.7%	95	20.2%	86	15.8%		
		5 or more	328	5.9%	612	11.5%	80	17.0%	95	17.5%		
	Drunkenness frequency											
		Never	**4237**	**75.6%**	**2911**	**54.7%**	**190**	**40.2%**	**260**	**63.6%**	840.30	<0.001
		1 or 2	952	17.0%	1422	26.7%	130	27.5%	142	22.1%		
		3 to 9	300	5.4%	687	12.9%	98	20.7%	80	9.8%		
		10 or more times	113	2.0%	302	5.7%	55	11.6%	67	4.5%		

Note: **Bold**—prevalent subgroup.

### 3.4. Family Member Drunkenness Exposure and Adolescent Drinking Patterns

Analysis of adolescent drinking patterns (drinking frequency, drinking quantity, drunkenness frequency) showed statistically significant (*p* < 0.001) differences between all levels of family member drunkenness exposure (Table 3). Analysis of associations between family member drunkenness exposure and adolescent drinking patterns revealed that in families where adolescents have never seen their family member drunk their drinking frequency, drinking quantity and drunkenness frequency are statistically significantly (*p* < 0.001) lower than in families where they have observed family member drunkenness sometimes or more (Table 3).

We found that in families where adolescents see their family member drunk sometimes (OR = 1.957) or once a month (OR = 3.457) or once a week or more often (OR = 2.611), the odds for adolescents to drink frequently were statistically significantly (*p* < 0.001) greater, compared to families where adolescents witnessed no drunkenness among family members (Table 2). Further analysis of other adolescent drinking pattern categories (drinking quantity, drunkenness frequency) revealed the same tendencies of family member drunkenness exposure impact on adolescent drinking patterns (Table 2). More detailed analysis showed that there is no statistically significant (*p* < 0.05) difference in adolescent drinking patterns (drinking frequency *p* = 0.744, drinking quantity *p* = 0.379, drunkenness frequency *p* = 0.540) percentages whether an adolescent sees family member drunk once a month (n = 476) or once a week (n = 321).

## 4. Discussion and Conclusions

This research gives a confident large-scale overview of which subgroups of adolescents depending on their family structure are at heightened risk of alcohol abuse. This study investigated adolescents’ alcohol consumption patterns depending on the family structure, and also the impact of a family member drunkenness exposure on adolescents’ alcohol consumption pattern.

The descriptive analysis of the study based on the participating 11 counties showed that out of 12,115 investigated adolescents approximately one third reported never drinking alcohol and two thirds reported drinking alcohol at least once a month. Slightly more than half of adolescents reported having seen their family member drunk sometimes or more often and less than half reported having never seen their family member drunk. The data revealed that approximately one fifth of 15 year old adolescents drink alcohol 2 to 4 times a month which corresponds with findings from the HBSC (2005, 2006) study [22].

The results showed that living in a family with both birth parents is protective factor against alcohol consumption in adolescence—adolescents from single parent families and step parent families tend to drink more than adolescents from both parent families [46]. This statement goes for the frequency of alcohol consumption, for the number of drinks on a typical day of drinking, and also for the frequency of adolescents being really drunk. Adolescents from single parent families have been shown to be at risk of psychiatric disorders and substance abuse [58,59]. Bifulco *et al*. [58] have also described a developmental model of disorders in which parental problems and experience of abuse/neglect in childhood combine with problems in school and with peers in adolescence, increasing the risk for substance use disorders in early adulthood. The results indicated no statistically significant differences between single parent families and step parent families regarding adolescents’ drinking patterns.

An essential finding of the current study is evident association between adolescents’ alcohol consumption pattern and their familial drunkenness exposure—whether the adolescents have ever seen a family member when they are drunk. If an adolescent has been exposed to family member drunkenness, the odds are significantly higher that they drink any alcohol, that they drink alcohol in bigger quantities, and also that they have been drunk themselves [18]. The association is particularly strong between family member drunkenness exposure and adolescent’s drunkenness frequency. It is noteworthy that regardless how often an adolescent has seen his/her family member drunk (sometimes, once a month, once a week or more often), merely the fact that it has happened increases the odds to have harmful alcohol consumption patterns. These results attest the influence of social learning theory [60] in today’s world and the importance of family members’ exemplar behavior patterns to adolescent behavior [48,52]. Drawing upon transactional developmental theories Patterson and colleagues [61] have shown that the risk for behavioral and mental health problems of adolescents stem from the parents not teaching their children appropriate forms of social interaction and problem solving which in turn act as a cascading factor for development of subsequent antisocial behavior and substance abuse problems. The findings can be also interpreted through attachment theory—where conflict and lack of support in close relationships increase the risk of adolescent emotional disorders, low self-esteem and the possible onset of substance abuse. Childhood adversity is strongly associated with the attachment styles within the family which in turn contributes in the recurrence of similar behavior patterns in the adolescents’ future relationships [62,63].

The study has several limitations and also implications for future research. The data used for this research was cross-sectional and enabled to investigate the differences between different family type patterns as reported by respondents. The contemporary family structure diversity makes it hard to distinguish real single parent families from other types of cohabitation [64]. Also no adjustment was made for a range of factors that could be relevant to the association between family structure, exposure to family drunkenness and adolescent drinking, for example depression, anxiety or adolescent wellbeing. The study does not give an overview of possible dynamics before “change process” from one family type into another—or ongoing disruptions or disturbances in the family. It can be hypothesized that maybe some step parent families have experienced significant change during the initial family break up and increase thereby the risk to adolescent drinking problems [33]. Step parent and single parent families could be investigated more thoroughly and separately to see the impact of change itself to adolescent drinking habits. Also what needs to be further investigated are the familial, parental and psycho-social protective factors within different family types that might influence adolescents’ drinking less than the groups’ average.
